# A Foreword to Workers for the Sick!

**Published:** 1911-12-23

**Authors:** John L. Griffiths

**Affiliations:** Consul-General for the United States.


### A FOREWORD TO WORKERS FOR THE SICK!

By the HONOURABLE JOHN L. GRIFFITHS, Consul-General for the United States.

Being an Address to the League of Mercy at St. James's Palace.

At this season of the year, when the Yule log glows and the world is being hung with holly, and the loved ones gather from near and from far and young voices and merry laughter are heard about the hearth, and the old recapture their youth in an infectious atmosphere of gaiety and good cheer, and the only thing that mars a husband's contentment is the anticipation of the personal gift that he will receive from his wife on Christmas morning, and which she will use herself for ever afterwards. At this joyous time, when all the bells peal out "Peace on earth, good will to men," we certainly should not be so selfish in our happiness as not to wish to share it with those who are less fortunate than ourselves. An American philosopher has remarked that everybody says it is more blessed to give than to receive, but that nobody ever says it is easier. This philosopher, however, was mistaken, as philosophers often have been, for nothing is more encouraging in the social work of to-day than the fact that so many men and women—an increasing number each year—realise that it is more blessed to give than to receive. It is the realisation of this fact in England which makes it possible to maintain the multitudinous charities and philanthropies which are such a crown of glory of your Empire.

### Once Unfrequented now a Broad Highway.

It was said of John Howard that "he trod an open but unfrequented path to immortality." That lonely path, we rejoice to know, is rapidly becoming a broad highway and many are they who walk therein. The pessimist never renders any real service to mankind, for pessimism, we must remember, is a very different thing from criticism. We will never help our fellow men unless we believe in them, and in their desire and capacity for improvement. One of the reasons we do not do more for those who need our help is that we do not come sufficiently in touch with their necessities. To many the exigencies of the poor in London seem as remote as an earthquake in Messina, a famine in India, the foundering of a ship in the Black Sea, or the collapse of a mine in the Ural Mountains. We read about these grim happenings and our emotions are stirred, our sympathies are excited, but our conduct is not influenced, and we pass quickly on to the perusal of the latest art criticism or book review, or to something utterly trivial.

### The Final Test of Civilisation.

Only those who really go among the poor can know the vital character of the work that "The League of Mercy" is doing. Read "Over the Bridges" if you want to realise how far removed the West End is from the East. It seems to be true right here in London that "East and West are twain, and never the two shall meet." At least, it would be true if it were not for the angels of mercy and light and love who are constantly narrowing the chasm as they go about their errands of good will. What is needed is not curiosity, but intelligence, knowledge, sympathy, and effort when we are dealing with the great problems of poverty and suffering. The final test of civilisation is the regard shown for the weak and poor and unfortunate. Whatever the resources of a nation may be, however glorious its literature, however splendid its art and architecture, however wise its laws, if it be wanting in the practice of the humanities, it is a moral failure.

### The Development of the Heart.

The development of the heart, if I may so describe it, is the most remarkable thing that has happened in the last fifty years—more marvellous even than the transforming discoveries of science. It has been manifested in a myriad beautiful ways—in the gathering of neglected children into the warm arms of love, in the establishment of homes where the poor and weary may rest, in the protection of birds and animals from both thoughtless and intentional cruelty, and in the building of hospitals where even the poorest receive without cost the same care and attention for which the well-to-do pay so large a price. The modern hospital sprang from the Sermon on the Mount, from the democracy of the teachings of the Founder of our faith. Edward VII. is often spoken of as the Great Peacemaker, as the ruler who did so much to remove international misunderstandings and jealousies, and to advance international friendships. But does he not deserve a still higher title, that of the "Lover of man," because of the profound interest he took in the organisation and administration of the charities of his realm. "The League of Mercy," I believe, was his thought. If not his thought, it was greatly in his thought, and will always be associated with his name.

### Why "The League of Mercy" Appeals to Me!

In speaking a short time ago at a meeting of "The League of Mercy" I mentioned several reasons why the work of the League appealed to me. May I add one or two additional reasons this afternoon? It appeals to me because hospitals are strong bonds of union between the various countries. The physicians of America, for example, come to the London hospitals to learn, and the English physicians in turn visit the American hospitals to see how they are conducted, and through this interchange they gain not only much in professional knowledge but also in human sympathy. The hospitals pay no attention to class or race, to sect or creed. They do not ask if a patient is an Anglican or a Catholic, a Presbyterian, or a Methodist, or a Congregationalist, a Jew or a Gentile, a Mohammedan or a Hindoo—whether he be black or brown, yellow or white, they simply ask if he is ill, and straightway try, if possible, to heal him. The hospitals of the world are fine expressions of the oneness of mankind. London hospitals do not limit their activities to this great social organism, this "dreadful, delightful," bewildering, fascinating, coercing city, which, for convenience, is called London, for a very considerable portion of their patients come from out of town. A thought that is uppermost in my mind to-day is that the members of "The League of Mercy" are serving without any desire of compensation. The annual dividend the League pays to the hospitals is practically its entire capital stock, which is gathered afresh each year. I am interested, too, in the manner in which the funds are accumulated, not usually by thousands of pounds from wealthy subscribers, but ordinarily by shillings and pence contributed by those who often have to make a sacrifice in order to give their mite. The gift is greatly enhanced in value because of the element of sacrifice, because of the self-denial which has to be practised. The persons who have been so graciously decorated with the "Order of Mercy" by her Royal Highness this afternoon have won a great distinction. The decorations which have been bestowed show that suffering men and women have been relieved, and that we recognise that a heart overflowing with sympathy and compassion and love is the most priceless treasure in the world.

An Example from Active Service.

During the Atlanta campaign in the American Civil War President Harrison said: "The marching and fighting had been largely in the brush. Sometimes in advance the commander of a regiment could see no more than half of his own line, while the supports to his right and left were wholly hidden. To him it seemed as if his battalion was making an unsupported assault. The extended lines, the reserves, were matters of faith. But one day the advancing army broke suddenly from the brush into a savannah, a long, narrow, natural meadow, and the army was revealed. From the centre, far to the right and left division, brigade, and regimental colours appeared, and associated with each of these was the one flag that made the army one. A view of the whole army now and again is a good thing."

A Vision that Quickens the Heart.

The vision quickens the heart and strengthens the will. At these annual gatherings the separated branches of the "League of Mercy" realise that while they are working seemingly at times in an isolated and detached way, they are nevertheless parts of a great army working for a common object as they endeavour to make the men and women for whose welfare they strive strong-limbed, clear-minded, pure-hearted and pure-spirited, and capable of fulfilling a high destiny. Although the immediate results of your labours may seem small, the promise is glorious and infinite. If anyone desires to know more about the work of "The League of Mercy" I would simply say, "Go, search the simple annals of the poor," for there you will find the record written in terms of tenderness, and gratitude, and love—in terms of life itself.

